# Solid Biomass Fuel Use and Respiratory Health Among Women and Children in South Asian Countries: A Systematic Review and Meta-Analysis

**DOI:** 10.7759/cureus.104012

**Published:** 2026-02-21

**Authors:** Kulumina Dash, Sasmita Nayak, Kripalini Patel, Milinda Mishra, Pratap K Jena, Krushna Chandra Sahoo

**Affiliations:** 1 Public Health, Kalinga Institute of Industrial Technology (KIIT) Deemed to be University, Bhubaneswar, IND; 2 Nursing Sciences, Kalinga Institute of Industrial Technology (KIIT) Deemed to be University, Bhubaneswar, IND; 3 Public Health, Indian Council of Medical Research-Regional Medical Research Centre, Bhubaneswar, IND; 4 Health Promotion, South Asian Institute of Health Promotion (SAIHP), Bhubaneswar, IND; 5 Health Care Management, Swiss School of Business and Management (SSBM) Geneva, Geneva, CHE

**Keywords:** biomass fuel, children, respiratory health, south asia, systematic review, women

## Abstract

The use of biofuels for cooking in households has been associated with an increased risk of respiratory infections, particularly among women and children. However, there remains a dearth of systematic evidence regarding the respiratory health impacts of biofuel use in South Asian countries. This review aimed to compare the prevalence of respiratory infections among women and children under five years old in households using unimproved cooking fuels versus those using improved alternatives. Articles were systematically retrieved from the PubMed, Embase, and Web of Science databases. Pooled prevalence estimates were calculated through meta-analysis utilizing the random-effects model. From a pool of 4,190 identified articles, 86 full-text articles were scrutinized, leading to the inclusion of 36 studies. Analysis revealed a notably higher prevalence of respiratory infections among individuals utilizing unimproved cooking fuels, with 23.3% prevalence among women compared to 8% among users of improved fuels. Women using unimproved fuels also exhibited higher rates of acute respiratory infections (29.2%) and chronic respiratory infections (17.1%) compared to those using improved fuels (11.2% and 5.3%, respectively). Likewise, children in households using unimproved fuels demonstrated a significantly higher prevalence of acute respiratory infections (26.4%) compared to those using improved fuels (11.8%). These findings underscore the urgent imperative to advocate for the adoption of improved cooking fuels and technologies in South Asian countries to mitigate the burden of respiratory infections.

## Introduction and background

Biomass fuel serves as the primary domestic energy source for nearly one-third of the global population, underscoring its widespread significance [1]. However, its utilization for household cooking is accompanied by a substantial contribution to indoor air pollution (IAP), affecting over four million people annually worldwide [2]. Approximately 2.4 billion individuals inhabit households reliant on solid biomass fuels for cooking and heating, with an additional 0.6 billion depending on coal [3]. Within these households, levels of PM10 and PM2.5 often surpass recommended guidelines for mean 24-hour concentration, with cooking activities exacerbating these hazardous levels [4].

Beyond statistical figures, the ramifications of biomass fuel dependence are profound. IAP emerges as a significant environmental risk factor, accounting for 5% of all women's deaths annually in developing countries due to respiratory infections [5]. Projections from the International Energy Agency (IEA) forecast that by 2030, one billion individuals will still lack access to electricity, while 2.6 billion will continue to lack access to clean cooking fuel [6]. Presently, approximately 2.5 billion people worldwide rely on biomass fuels for cooking, heating, and lighting, including materials like manure, wood, agricultural residues, and coal [7]. This reliance is particularly pronounced in Africa, where over 500 million people (78% of the population) employ biomass fuel for cooking and heating [8,9]. Similarly, South Asia, encompassing countries such as India, Bangladesh, Pakistan, Nepal, Sri Lanka, and Bhutan, witnesses extensive use of biomass fuels like wood, crop residues, and dung, particularly in rural settings [10-13].

Moreover, mounting evidence establishes a troubling association between biomass fuel exposure and respiratory ailments such as tuberculosis, chronic obstructive pulmonary disease (COPD), and lung cancer, significantly contributing to the global disease burden [13]. Women and children under five emerge as particularly vulnerable to IAP from these fuels, given their prolonged exposure near cooking fires [13]. In South Asia, where women traditionally assume household chores and cooking duties, they endure disproportionate exposure to IAP's detrimental effects due to extended proximity to smoke. Furthermore, commonly used biomass fuels emit various organic compounds such as benzene, formaldehyde, 1,3-butadiene, and polyaromatic hydrocarbons, further exacerbating health risks [14,15]. Notably, studies in India have linked IAP to a spectrum of health conditions, including 20% of ischemic heart disease (IHD), 23% of strokes, 45% of COPD cases, 21% of lung cancer cases, and 22% of acute respiratory tract infections (ARI) [15].

In South Asia, the confluence of household air pollution and biomass fuel utilization poses intricate and substantial health risks to women [16]. Given their primary role in cooking, women are predisposed to heightened exposure to household air contaminants. Furthermore, households reliant on biofuels are at an increased risk of respiratory infections. The predominant role of women in South Asia, revolving around cooking responsibilities, exacerbates this issue due to their prolonged proximity to pollution sources. Despite these realities, a systematic understanding of the health impacts of biofuel use on women and children in South Asian countries remains lacking. Hence, this comprehensive systematic review and meta-analysis aim to elucidate the health effects of solid biomass fuels on women and children under five in South Asian countries, seeking to inform policy changes and address this urgent concern.

## Review

Methods

This systematic review was conducted according to the Preferred Reporting Items for Systematic reviews and Meta-Analyses (PRISMA) guideline and registered in PROSPERO (http://www.crd.york.ac.uk/PROSPERO/) (CRD42021260063).

Databases and Search Strategies

We conducted independent searches on three online databases: PubMed, Embase, and Web of Science. To create a comprehensive search strategy, we used relevant keywords and MeSH terminology related to “Household Air Pollution” and “Respiratory Health Outcomes.” This strategy encompassed three major domains: “Household Air Pollution,” “Respiratory Health Outcomes,” and “South Asia.” We combined these domains and terms using the Boolean operator "AND” to create custom search queries tailored to each database's requirements and specifications.

Inclusion Criteria

The review centered on examining acute and chronic respiratory infections in women and children under the age of five within households that relied on unimproved (solid biomass) cooking fuels in South Asian countries, comparing them to households that used improved cooking fuels. We considered studies published in English between January 2010 and June 2021 for inclusion. The selected study types encompassed cross-sectional, case-control, cohort, longitudinal, case-cross-over, and intervention studies conducted in the South Asian region. Excluded from the review were editorial reports, review articles, and case reports.

Screening and Identification of Studies

Upon identifying all articles through searches, we imported them into reference management software (EndNote X8, Clarivate, London, UK) to remove duplicate entries. Following this, the records were transitioned to Rayyan (Rayyan Systems Inc., Cambridge, MA, US), where the initial screening involved reviewing titles and abstracts. The primary screening was carried out by three independent researchers. Subsequently, potentially pertinent studies underwent a comprehensive eligibility assessment during a full-text screening phase. Any discrepancies or disagreements among the authors were addressed through open discussion and mutual consensus.

Quality Assessment, Data Extraction, and Synthesis

This study employed the Joanna Briggs Institute (JBI) critical appraisal checklist [17], tailored for analytical cross-sectional studies, to evaluate the methodological rigor of the studies incorporated in this analysis. For the systematic data extraction of pertinent study details and outcomes, we employed a pre-designed and standardized data extraction form. Two authors autonomously collected relevant information using a Microsoft Excel spreadsheet (Microsoft Corporation, Redmond, WA, US), ensuring consistency and accuracy. Subsequently, the extracted data were cross-validated by another author to enhance reliability. The extraction parameters included information about the authors, the study design, the study location, the sample size, the publication year, the study period, the number of households that used improved and unimproved cooking fuels, and the number of acute respiratory and chronic infections among women and children under the age of five.

The meta-analysis was performed using a random-effects model to determine pooled prevalence among different subgroups. The models estimated the overall effect size and assessed study heterogeneity. Subgroup analysis and meta-regression were used to investigate sources of heterogeneity and evaluate how different study characteristics may influence the results. The results are presented in forest plots to help interpret the results by visualizing individual study effect sizes and the combined effect size. The funnel plots are used to determine whether or not there is publication bias.

Results

**Figure 1:**
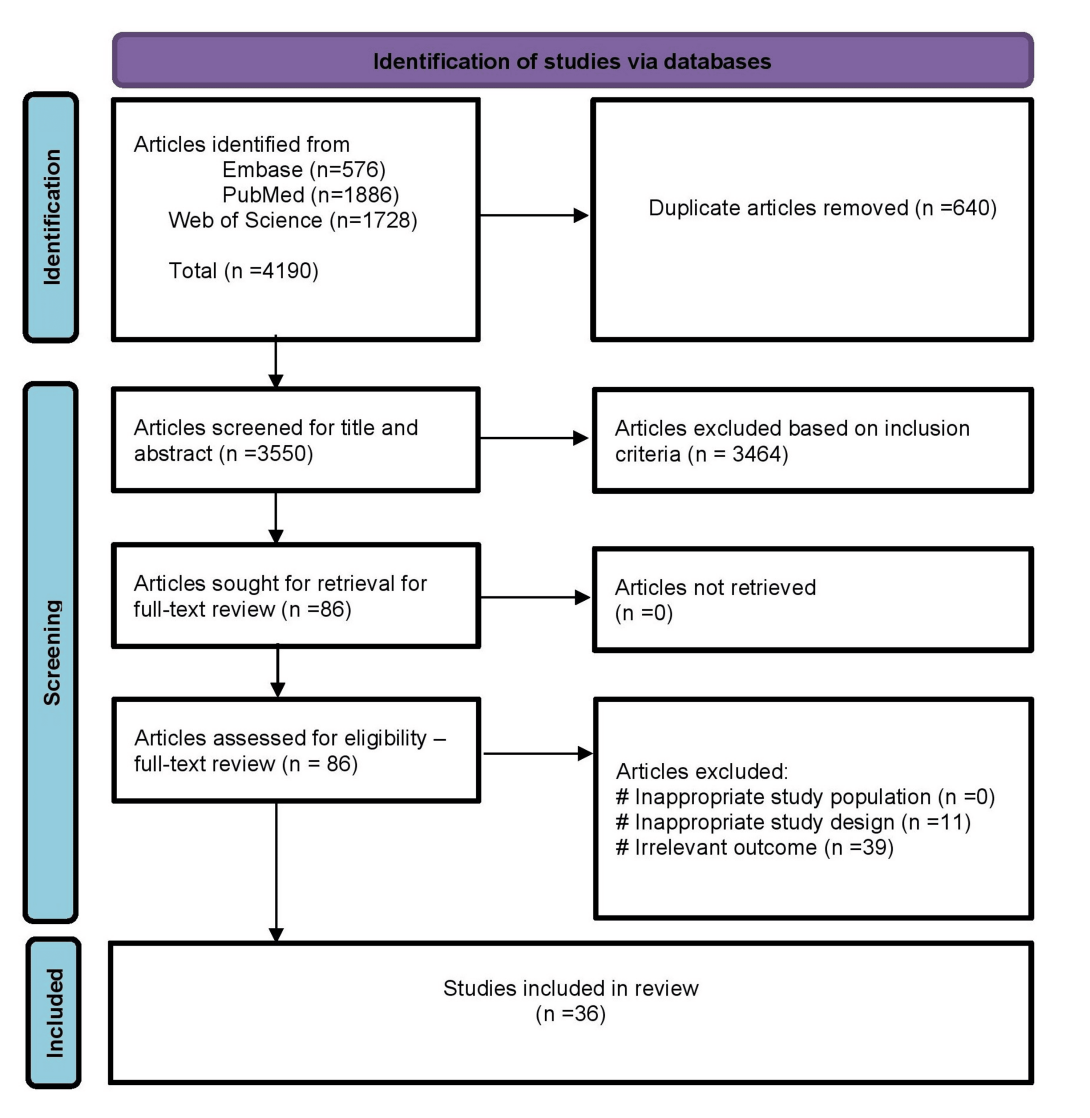
PRISMA flow diagram. PRISMA: Preferred Reporting Items for Systematic reviews and Meta-Analyses

The initial search retrieved a total of 4,190 articles, which were subsequently reduced to 3,550 after eliminating duplicates. Following a meticulous review of titles and abstracts, 3,464 articles were excluded, primarily due to the study area being outside of South Asia or not aligning with the expected outcome. This left us with 86 articles that underwent a comprehensive full-text review. Ultimately, 36 studies were identified as meeting the inclusion criteria. For a visual representation of this process, please refer to Figure 1 in the detailed PRISMA flow diagram.

The systematic review encompassed a total of 36 articles, with 21 focusing on women and 15 on children. Tables 1, 2, respectively, present detailed characteristics of the women participants and households with children under the age of five.

**Table 1:** Characteristics of women participants.

Author, year	State	Country	Study design	Settings	Age in years	Total women	Women with improved fuel	Women with unimproved fuel	Chronic respiratory infection among improved fuel users	Chronic respiratory infection among unimproved fuel users	Acute respiratory infection among improved fuel users	Acute respiratory infection in unimproved fuel users
James et al., 2020 [14]	Manipal	India	Community-based cross-sectional	Rural	18+	632	66	566	Not available	Not available	10	268
Parikh et al., 2016 [15]	Rajasthan	India	Large survey	Rural	15+	41,965	1,259	40,706	12,589	29,366	6	Not available
Panigrahi and Padhi, 2018 [18]	Odisha	India	Community-based cross-sectional	Rural	18-49	917	344	573	27	84	Not available	Not available
Guddattu et al., 2010 [19]	Not available	India	Large survey	Rural	15-49	85,699	34,870	50,829	551	839	Not available	Not available
Agrawal, 2012 [20]	Not available	India	Large survey	Both	20-49	124,385	37,067	87,318	630	1,746	Not available	Not available
Pathak et al., 2019 [21]	Uttar Pradesh	India	Community-based cross-sectional	Rural	18–40	310	140	170	Not available	Not available	50	106
Kaur-Sidhu et al., 2019 [22]	Punjab	India	Community-based cross-sectional	Rural	30-60	50	15	35	Not available	Not available	2	14
Sukhsohale et al., 2012 [23]	Nagpur	India	Community-based cross-sectional	Rural	15+	535	283	252	4	9	42	59
Sukhsohale et al., 2013 [24]	Nagpur	India	Community-based cross-sectional	Rural	15+	444	192	252	26	52	5	12
Dutta and Ray, 2014 [25]	West Bengal	India	Community-based cross-sectional	Rural	22-41	480	236	244	10	40	45	139
Mukherjee et al., 2014 [26]	West Bengal	India	Community-based cross-sectional	Rural	23–43	1,119	438	681	13	57	125	347
Alim et al., 2014 [27]	Bogra	Bangladesh	Community-based cross-sectional	Rural	15-58	224	Not available	224	Not available	152	Not available	67
				Urban	15-58	196	196	Not available	63	Not available	22	Not available
Pial et al., 2020 [28]	Sylhet	Bangladesh	Community-based cross-sectional	Rural	18+	510	Not available	255	Not available	3	Not available	17
				Urban	18+	510	255	Not available	20	Not available	4	Not available
Rabha et al., 2019 [29]	Assam	India	Community-based cross-sectional	Rural	18–55	266	Not available	266	Not available	9	Not available	17
				Urban	18–55	82	82	Not available	2	Not available	5	Not available
Arora et al., 2018 [30]	Delhi	India	Community-based cross-sectional	Urban	15-59	500	Not available	500	Not available	Not available	Not available	124
Mondal and Chakraborty, 2015 [31]	West Bengal	India	Community-based cross-sectional	Rural	26-39	100	Not available	100	Not available	65	Not available	5
Bolla et al., 2021 [32]	Tamilnadu	India	Community-based cross-sectional	Rural	18+	100	Not available	100	Not available	Not available	Not available	19
Medgyesi et al., 2017 [33]	Dhaka	Bangladesh	Community-based cross-sectional	Rural	18-65	15	Not available	15	Not available	Not available	Not available	12
Pratali et al., 2019 [34]	Khumbu	Nepal	Case-control	Rural	16-75	51	Not available	51	Not available	4	Not available	Not available
Adhikari et al., 2020 [35]	Western Nepal	Nepal	Community-based cross-sectional	Urban	40+	661	424	237	Not available	228	Not available	Not available
Johnson et al., 2011 [36]	Tamilnadu	India	Community-based cross-sectional	Rural	30+	900	147	753	Not available	Not available	Not available	Not available

**Table 2:** Characteristics of households having children.

Authors, year	Country	Study design	Settings	Total children	Age in years	Children with improved cooking fuel	Improved acute respiratory infection	Children with unimproved cooking fuel	Unimproved acute respiratory infection
Khalequzzaman et al., 2011 [37]	Bangladesh	Cross-sectional	Both	102	0-5	Not available	Not available	102	89
Nasanen-Gilmore et al., 2015 [38]	Bangladesh	Cross-sectional	Rural	321	0-5	Not available	Not available	318	63
Bates et al., 2013 [39]	Nepal	Case-control	Urban	917	2-3	457	201	460	251
Ramesh Bhat et al., 2012 [40]	India	Case-control	Rural	202	0-5	69	6	133	95
Khan and Lohano, 2018 [41]	Pakistan	Cross-sectional	Both	11,040	0-5	3,533	971	7,507	1,457
Acharya et al., 2015 [42]	Nepal	Cross-sectional	Both	4,802	0-5	741	25	4,061	197
Mandal et al., 2020 [43]	India	Cross-sectional	Both	247,743	0-5	88,667	2,172	159,075	4,613
Arlington et al., 2019 [44]	India	Prospective	Rural	1,586	0-0.5	499	66	1,087	236
Hasan et al., 2019 [45]	Bangladesh	Cross-sectional	Urban	10,575	0-5	6,402	1,223	4,173	938
Karki et al., 2014 [46]	Nepal	Case-control	Both	200	0-5	138	31	62	19
Mondal and Paul, 2020 [47]	India	Cross-sectional	Both	247,743	0-5	86,225	2,069	161,528	4,684
Nirmolia et al., 2018 [48]	India	Cross-sectional	Urban	624	1-5	190	5	434	97
Patel et al., 2019 [49]	India	Cross-sectional	Both	932,341	0-5	125,231	10,379	807,110	300,645
Rana et al., 2019 [50]	Afghanistan	Cross-sectional	Both	27,565	0-5	6,799	1,033	20,766	3,883
Upadhyay et al., 2015 [51]	India	Cohort	Both	3,961	0-5	1,030	102	2,931	542

Regarding the women's studies [14,15,18-36], the majority consisted of community-based cross-sectional studies, while three were large-scale investigations, and one adopted a case-control study design. Predominantly, these studies were conducted in India, with an additional five from Bangladesh and two from Nepal. Most of the research was concentrated in rural areas, although four studies extended to both rural and urban settings, while only one study was exclusively conducted in urban areas.

Table 3 shows that all listed studies meet the JBI critical appraisal checklist for analytical cross-sectional studies, demonstrating comprehensive methodological quality across all evaluated domains. The study's inclusion, description of subjects, exposure measurement, objective criteria, identification of confounding factors, strategies to address them, outcome measurement validity, and appropriate statistical analysis are marked "yes." If further nuance is needed, per-study specifics or qualitative synthesis can be provided.

**Table 3:** Assessment of methodological quality using JBI critical appraisal checklist for analytical cross-sectional studies. JBI: Joanna Briggs Institute

Author, year	Inclusion in the sample clearly defined	Study subjects and the setting described in detail	Exposure measured in a valid and reliable way	Objective, standard criteria used for measurement of the condition	Confounding factors identified	Strategies to deal with confounding factors	Outcomes measured in a valid and reliable way	Appropriate statistical analysis
James et al., 2020 [14]	Yes	Yes	Yes	Yes	Yes	Yes	Yes	Yes
Parikh et al., 2016 [15]	Yes	Yes	Yes	Yes	Yes	Yes	Yes	Yes
Panigrahi and Padhi, 2018 [18]	Yes	Yes	Yes	Yes	Yes	Yes	Yes	Yes
Guddattu et al., 2010 [19]	Yes	Yes	Yes	Yes	Yes	Yes	Yes	Yes
Agrawal, 2012 [20]	Yes	Yes	Yes	Yes	Yes	Yes	Yes	Yes
Pathak et al., 2019 [21]	Yes	Yes	Yes	Yes	Yes	Yes	Yes	Yes
Kaur-Sidhu et al., 2019 [22]	Yes	Yes	Yes	Yes	Yes	Yes	Yes	Yes
Sukhsohale et al., 2012 [23]	Yes	Yes	Yes	Yes	Yes	Yes	Yes	Yes
Sukhsohale et al., 2013 [24]	Yes	Yes	Yes	Yes	Yes	Yes	Yes	Yes
Dutta and Ray, 2014 [25]	Yes	Yes	Yes	Yes	Yes	Yes	Yes	Yes
Mukherjee et al., 2014 [26]	Yes	Yes	Yes	Yes	Yes	Yes	Yes	Yes
Alim et al., 2014 [27]	Yes	Yes	Yes	Yes	Yes	Yes	Yes	Yes
Pial et al., 2020 [28]	Yes	Yes	Yes	Yes	Yes	Yes	Yes	Yes
Rabha et al., 2019 [29]	Yes	Yes	Yes	Yes	Yes	Yes	Yes	Yes
Arora et al., 2018 [30]	Yes	Yes	Yes	Yes	Yes	Yes	Yes	Yes
Mondal and Chakraborty, 2015 [31]	Yes	Yes	Yes	Yes	Yes	Yes	Yes	Yes
Bolla et al., 2021 [32]	Yes	Yes	Yes	Yes	Yes	Yes	Yes	Yes
Medgyesi et al., 2017 [33]	Yes	Yes	Yes	Yes	Yes	Yes	Yes	Yes
Pratali et al., 2019 [34]	Yes	Yes	Yes	Yes	Yes	Yes	Yes	Yes
Adhikari et al., 2020 [35]	Yes	Yes	Yes	Yes	Yes	Yes	Yes	Yes
Johnson et al., 2011 [36]	Yes	Yes	Yes	Yes	Yes	Yes	Yes	Yes
Khalequzzaman et al., 2011 [37]	Yes	Yes	Yes	Yes	Yes	Yes	Yes	Yes
Nasanen-Gilmore et al., 2015 [38]	Yes	Yes	Yes	Yes	Yes	Yes	Yes	Yes
Bates et al., 2013 [39]	Yes	Yes	Yes	Yes	Yes	Yes	Yes	Yes
Ramesh Bhat et al., 2012 [40]	Yes	Yes	Yes	Yes	Yes	Yes	Yes	Yes
Khan and Lohano, 2018 [41]	Yes	Yes	Yes	Yes	Yes	Yes	Yes	Yes
Acharya et al., 2015 [42]	Yes	Yes	Yes	Yes	Yes	Yes	Yes	Yes
Mandal et al., 2020 [43]	Yes	Yes	Yes	Yes	Yes	Yes	Yes	Yes
Arlington et al., 2019 [44]	Yes	Yes	Yes	Yes	Yes	Yes	Yes	Yes
Hasan et al., 2019 [45]	Yes	Yes	Yes	Yes	Yes	Yes	Yes	Yes
Karki et al., 2014 [46]	Yes	Yes	Yes	Yes	Yes	Yes	Yes	Yes
Mondal and Paul, 2020 [47]	Yes	Yes	Yes	Yes	Yes	Yes	Yes	Yes
Nirmolia et al., 2018 [48]	Yes	Yes	Yes	Yes	Yes	Yes	Yes	Yes
Patel et al., 2019 [49]	Yes	Yes	Yes	Yes	Yes	Yes	Yes	Yes
Rana et al., 2019 [50]	Yes	Yes	Yes	Yes	Yes	Yes	Yes	Yes
Upadhyay et al., 2015 [51]	Yes	Yes	Yes	Yes	Yes	Yes	Yes	Yes

In the children's studies [37-51], the majority were cross-sectional, complemented by three case-control studies, one prospective study, and one cohort study. India featured prominently as the research location, with three studies in Nepal, three in Bangladesh, one in Pakistan, and one in Afghanistan. A significant portion of these studies encompassed both rural and urban areas, with three studies focusing exclusively on each of these settings.

Out of the 21 women's studies [14,15,18-36], five focused on the prevalence of respiratory infections among women who solely used unimproved cooking fuels, while 16 examined those who used both improved and unimproved fuels. Among women using improved fuels, three studies addressed chronic respiratory infections, four focused on acute respiratory infections, and seven explored both acute and chronic respiratory infections. Similarly, among women using unimproved fuels, five studies investigated chronic respiratory infections, six studied acute respiratory infections, and eight examined both acute and chronic respiratory infections. Among the 15 children's studies, only two examined the prevalence of acute respiratory infections in households using unimproved cooking fuel. The remaining 13 studies focused on acute respiratory infections in households that used both improved and unimproved cooking fuels.

**Figure 2:**
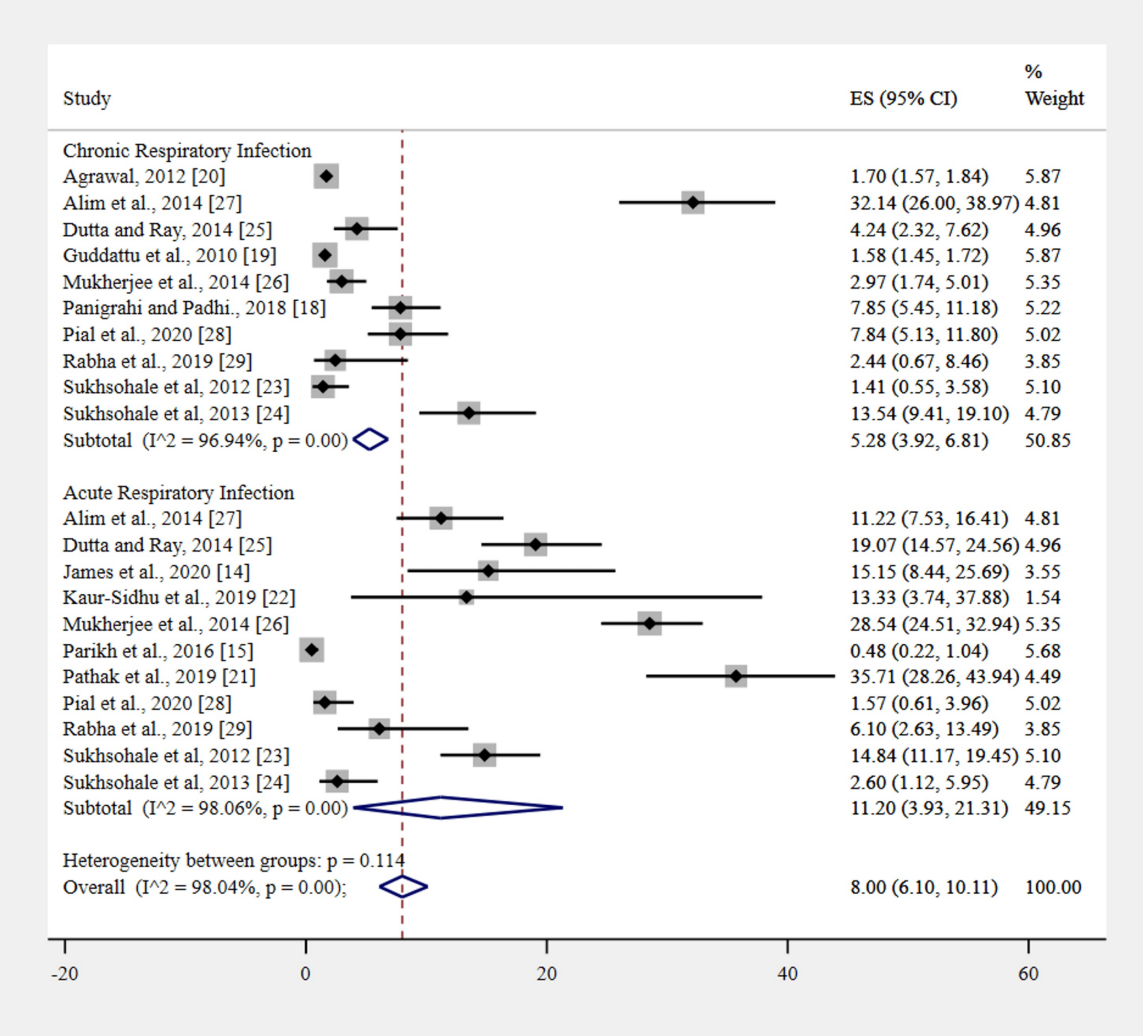
Acute and chronic respiratory infections in women who use improved cooking fuels.

The pooled prevalence of respiratory infections among women exposed to improved cooking fuel was 8% (95% CI 6.10-10.11; I² = 98.04%, P < 0.001). A subgroup analysis, classified by the type of respiratory infection, revealed a notable difference. Acute respiratory infections were higher at 11.20% (95% CI 3.93-21.31; I² = 98.06%, P < 0.001) compared to chronic respiratory infections, which stood at 5.28% (95% CI 3.92-6.81; I² = 96.94%, P < 0.001). Figure 2 depicts the forest plot describing acute and chronic respiratory infections in women who use improved cooking fuels.

**Figure 3:**
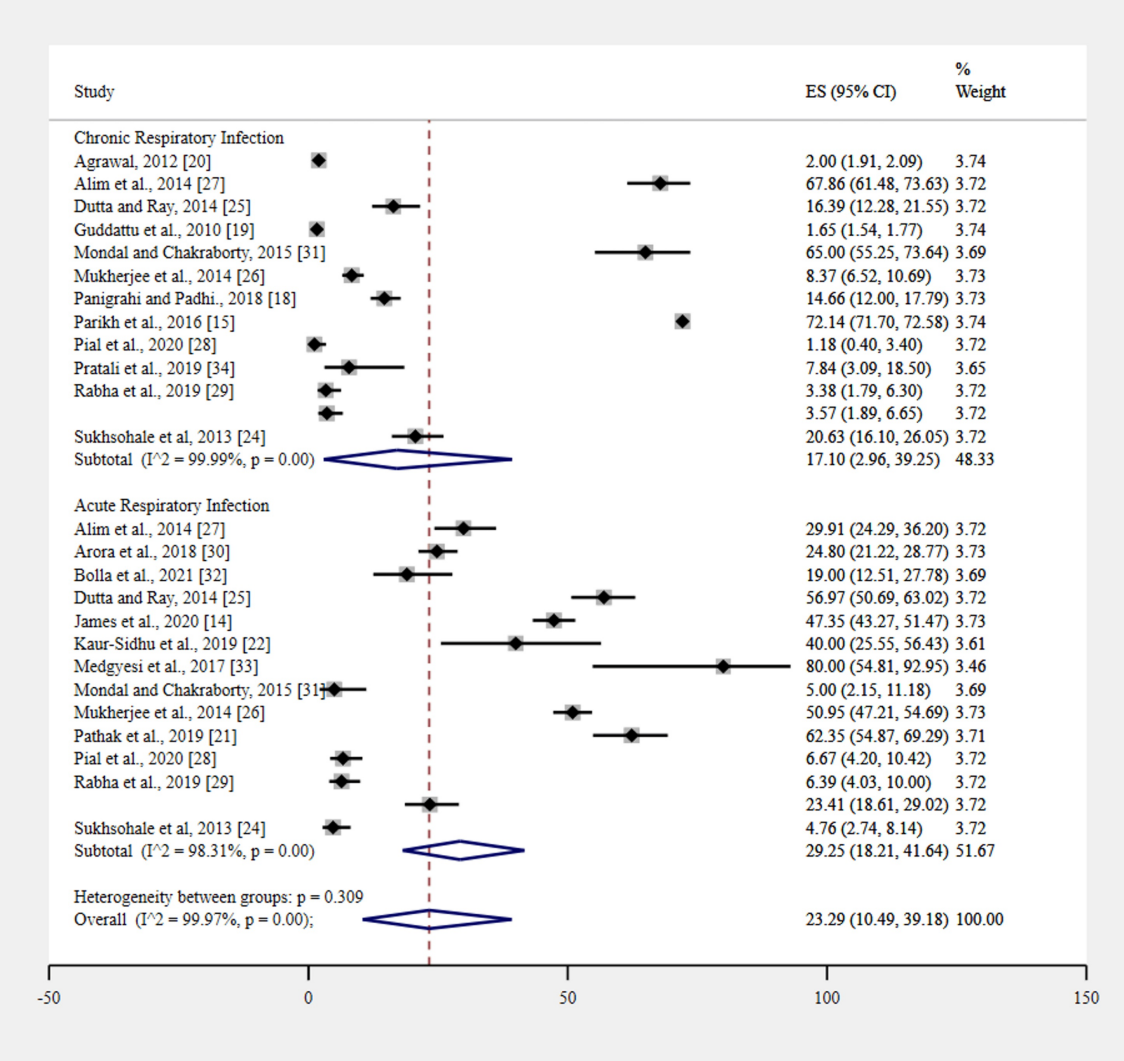
Acute and chronic respiratory infections in women who use unimproved cooking fuels.

The pooled prevalence of respiratory infections among women exposed to unimproved cooking fuel was 23.29% (95% CI 10.49-39.18; I² = 99.97%, P < 0.001). Subgroup analysis, categorized by the type of respiratory infection, revealed that the prevalence of acute respiratory infection was higher at 29.25% (95% CI 18.21-41.67; I² = 98.31%, P < 0.001) than chronic respiratory infection at 17.10% (95% CI 2.96-39.25; I² = 99.99%, P < 0.001). Figure 3 depicts the forest plot describing acute and chronic respiratory infections in women who use unimproved cooking fuels.

**Figure 4:**
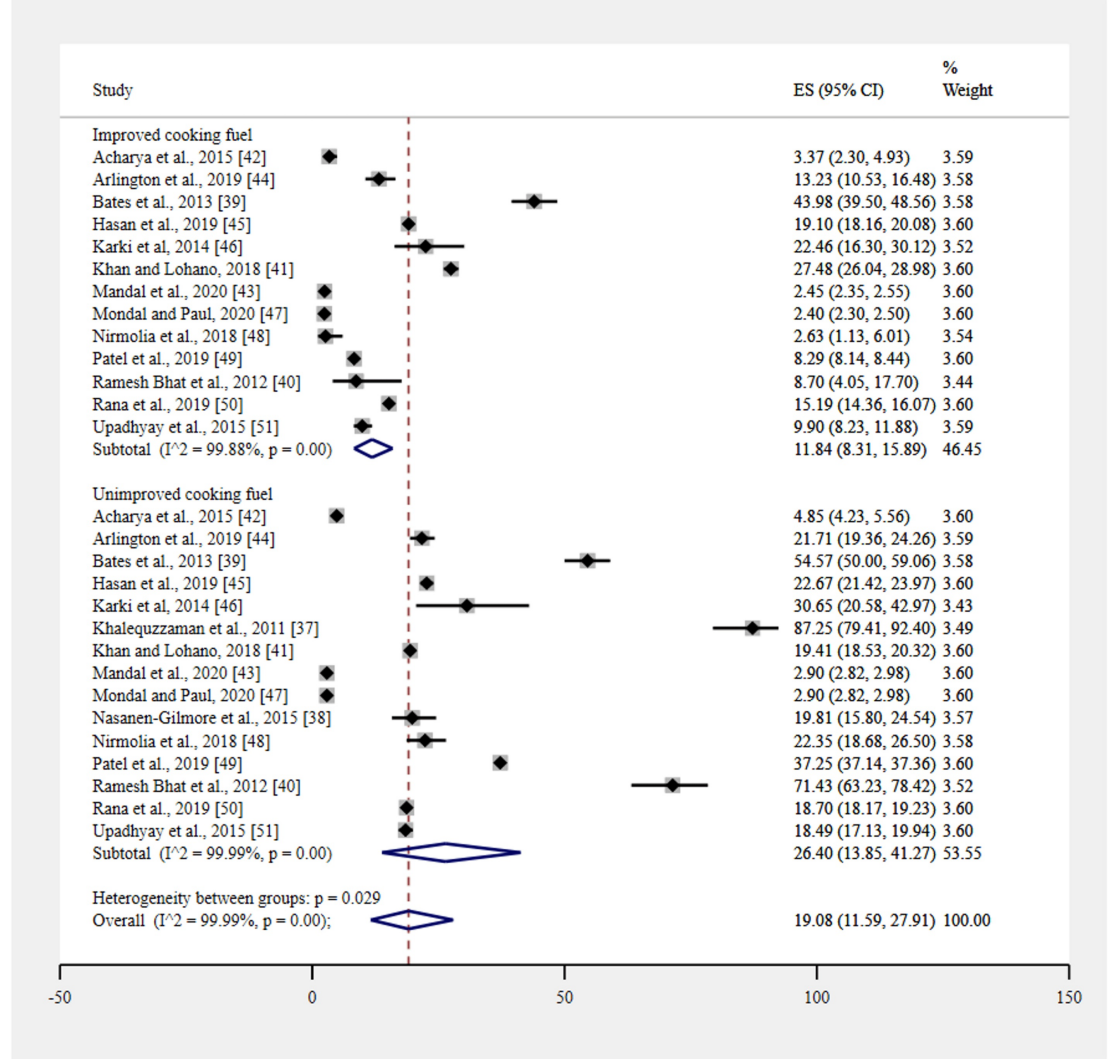
Acute respiratory infections in children with household cooking fuels (improved versus unimproved) using practices.

Figure 4 compares the prevalence of acute respiratory infections among children [37-51] living in households with and without improved cooking fuel practices. The overall prevalence was 19.08% (95% CI 11.59-27.91). The subgroup analysis revealed that the pooled prevalence was higher among children whose households used unimproved cooking fuel (26.40%, 95% CI 13.85-41.27) than among those whose households used improved cooking fuel (11.84%, 95% CI 8.31-15.54).

**Figure 5:**
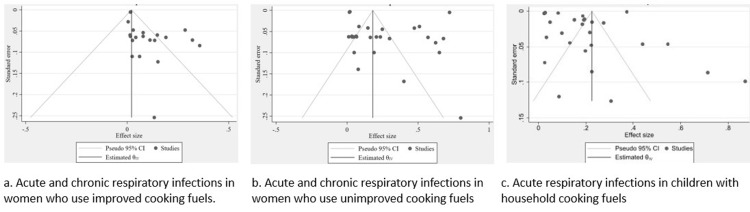
(a-c) Funnel plot showing publication bias.

The publication bias of the results is presented in a funnel plot (Figure 5). Figure 5a shows acute and chronic respiratory infections in women who use improved cooking fuels, where the spread is moderate, indicating reasonable heterogeneity but not extreme. Figure 5b shows acute and chronic respiratory infections in women who use unimproved cooking fuels, where a wider dispersion of study estimates suggests greater heterogeneity. Figure 5c shows acute respiratory infections in children with household cooking fuels, where the distribution shows some asymmetry with more studies on one side, which may hint at potential small-study effects or contextual differences specific to child populations. There is notable between-study heterogeneity across all groups, with greater dispersion in Figures 5b, 5c.

Discussion

The study identified a higher prevalence of respiratory infections among women and children in South Asia residing in households reliant on unimproved cooking fuel. Women exposed to unimproved fuel exhibited an increased likelihood of developing acute respiratory infections (29.2%) and chronic infections (17.1%). Similarly, a significant disparity in acute respiratory infections was observed among children. These findings underscore the imperative for strategic policy frameworks aimed at fostering the widespread adoption of clean cooking fuels, necessitating incentives, subsidies, and public awareness campaigns.

South Asia, grappling with the pervasive challenge of household air pollution stemming from cooking fuel usage and its associated respiratory infections, has witnessed concerted efforts through various initiatives. Notable among these is the Pradhan Mantri Ujjwala Yojana (PMUY), launched by the Indian government in May 2016. This scheme incentivizes the adoption of clean cooking fuels in rural India by subsidizing liquid petroleum gas (LPG) connections for rural households, which include a complimentary gas cylinder, regulator, and pipe [16]. In addition to the PMUY, similar programs have been implemented across other South Asian nations to advocate for cleaner cooking practices. The current implementation status of the PMUY in India demonstrates continued progress in expanding LPG access to rural and economically vulnerable households, with millions of new gas connections completed and a continued emphasis on beneficiary verification and policy refinements to prevent duplication and leakages. However, issues continue in guaranteeing a consistent fuel supply in remote areas, maintaining affordability in the face of growing LPG prices, and meeting ongoing demands for safe household energy practices and petrol cylinder safety. While administrative outreach and grievance redress mechanisms have improved, critical gaps in target accuracy, refill uptake consistency, and equitable benefits across states remain, highlighting the need for improved monitoring, strong supply chains, and complementary clean cooking energy initiatives to achieve universal clean cooking access.

In Bangladesh, the "Practical Action" program focuses on improving cookstoves and fosters community education about the benefits of cleaner cooking practices [52]. Meanwhile, Nepal's "National Improved Cook Stove Program" strives to replace traditional stoves with cleaner alternatives, emphasizing technological innovation and dissemination. Another noteworthy initiative in Nepal, the Urban Health Initiative (UHI), strives to mitigate household air pollution. In Sri Lanka, the "Sustainable Energy for All" (SE4ALL) initiative targets the reduction of household air pollution attributable to biomass fuel or unimproved fuels [53]. This initiative includes a component promoting clean cooking solutions, such as improved cookstoves and biogas systems, to diminish IAP and bolster health outcomes [54,55].

Nevertheless, challenges, including accessibility, affordability, behavioral change, and monitoring, impede the impact of PMUY and analogous programs in Bangladesh, Nepal, and Sri Lanka. Overcoming these hurdles is pivotal for the success of initiatives promoting clean cooking in South Asia. Addressing these challenges and fostering collaboration among governments, non-governmental organizations, and communities are essential for making substantial progress in reducing household air pollution and improving respiratory health. Investment in research and development holds promise for yielding efficient and affordable alternatives, thereby warranting further exploration of the impact of cleaner cooking fuels on health, the environment, and society.

Concurrently, there should be a prioritized focus on improving the accessibility of advanced cooking technologies, particularly in remote and marginalized areas. The effective implementation of policies necessitates robust monitoring and evaluation mechanisms [32-42]. A thorough, long-term assessment of intervention impacts on respiratory health outcomes and indoor air quality is crucial for informed decision-making and course adjustments. Collaborative efforts across various sectors, including health, energy, environment, and gender, are indispensable for the comprehensive implementation of policies. Coordinated actions between sectors can amplify the effectiveness of interventions and ensure a holistic approach to addressing the issue [44-47]. To effectively mitigate the grave threat of household air pollution in South Asia, comprehensive and effective policies are imperative, necessitating coordinated actions and investments among countries and extending policymakers' focus into additional sectors such as small manufacturing, agriculture, residential cooking, and waste management.

The study has inherent limitations that warrant consideration when interpreting results. Variability in study design, population, and outcome measures among the included studies may limit result generalizability. Unaccounted factors may influence respiratory infection risk in South Asian women and children. Nevertheless, the study's strengths lie in its comprehensive systematic review and meta-analysis, offering a robust summary of evidence on the link between solid biomass fuel use and respiratory health outcomes in South Asian countries. The findings provide valuable insights into the potential impact of interventions promoting improved cooking fuels and technologies on respiratory health outcomes.

## Conclusions

The findings underscore the urgent need to advocate for improved cooking fuels and technologies to mitigate the heightened risk of respiratory infections among women and children in South Asian nations. It implies that prioritizing interventions aimed at promoting cleaner cooking fuels and technologies is imperative for enhancing respiratory health outcomes in these communities. Public health authorities in South Asian countries must prioritize such interventions to effectively reduce the prevalence of respiratory infections and safeguard the health of women and children.

## References

[1] Kim KH, Jahan SA, Kabir E (2011). A review of diseases associated with household air pollution due to the use of biomass fuels. J Hazard Mater.

[2] Forum of International Respiratory Societies (2017). The Global Impact of Respiratory Disease. https://static.physoc.org/app/uploads/2019/04/22192917/The_Global_Impact_of_Respiratory_Disease.pdf.

[3] Po JY, FitzGerald JM, Carlsten C (2011). Respiratory disease associated with solid biomass fuel exposure in rural women and children: systematic review and meta-analysis. Thorax.

[4] Bruce N, Perez-Padilla R, Albalak R (2000). Indoor air pollution in developing countries: a major environmental and public health challenge. Bull World Health Organ.

[5] Bonjour S, Adair-Rohani H, Wolf J (2013). Solid fuel use for household cooking: country and regional estimates for 1980-2010. Environ Health Perspect.

[6] Birol F (2008). World Energy Outlook. https://iea.blob.core.windows.net/assets/89d1f68c-f4bf-4597-805f-901cfa6ce889/weo2008.pdf.

[7] Rosenthal J, Quinn A, Grieshop AP, Pillarisetti A, Glass RI (2018). Clean cooking and the SDGs: integrated analytical approaches to guide energy interventions for health and environment goals. Energy Sustain Dev.

[8] Ekouevi K, Tuntivate V (2012). Household Energy Access for Cooking and Heating: Lessons Learned and the Way Forward. World Bank Publications.

[9] Dida GO, Lutta PO, Abuom PO, Mestrovic T, Anyona DN (2022). Factors predisposing women and children to indoor air pollution in rural villages, Western Kenya. Arch Public Health.

[10] Faizan MA, Thakur R (2019). Association between solid cooking fuels and respiratory disease across socio-demographic groups in India. J Health Pollut.

[11] (2017). Office of the Registrar General, India. Report on Medical Certification of Cause of Death 2015. https://censusindia.gov.in/nada/index.php/catalog/40068/download/43738/Annual_Report_on_MCCD_2015.pdf.

[12] Prasad R, Garg R, Gupta N (2021). Biomass fuel and lung diseases: an Indian perspective. Climate Change and Global Public Health.

[13] (2024). Ritchie H, Roser M. Indoor air pollution. https://ourworldindata.org/indoor-air-pollution.

[14] James BS, Shetty RS, Kamath A, Shetty A (2020). Household cooking fuel use and its health effects among rural women in southern India-a cross-sectional study. PLoS One.

[15] Parikh JK, Sharma A, Singh C, Neelakantan S (2016). Providing Clean Cooking Fuel in India: Challenges and Solutions. Winnipeg, Canada: International Institute.

[16] Gupta A, Vyas S, Hathi P, Khalid N, Srivastav N, Spears D, Coffey D (2020). Persistence of solid fuel use in rural north India. Econ Polit Wkly.

[17] (2025). JBI. Critical appraisal tools. https://jbi.global/critical-appraisal-tools.

[18] Panigrahi A, Padhi BK (2018). Chronic bronchitis and airflow obstruction is associated with household cooking fuel use among never-smoking women: a community-based cross-sectional study in Odisha, India. BMC Public Health.

[19] Guddattu V, Swathi A, Nair NS (2010). Household and environment factors associated with asthma among Indian women: a multilevel approach. J Asthma.

[20] Agrawal S (2012). Effect of indoor air pollution from biomass and solid fuel combustion on prevalence of self-reported asthma among adult men and women in India: findings from a nationwide large-scale cross-sectional survey. J Asthma.

[21] Pathak U, Kumar R, Suri TM, Suri JC, Gupta NC, Pathak S (2019). Impact of biomass fuel exposure from traditional stoves on lung functions in adult women of a rural Indian village. Lung India.

[22] Kaur-Sidhu M, Ravindra K, Mor S, John S, Aggarwal AN (2019). Respiratory health status of rural women exposed to liquefied petroleum gas and solid biomass fuel emissions. Air Soil Water Res.

[23] Sukhsohale ND, Narlawar UW, Ughade SN, Kulkarni H (2012). Even partial reduction of biomass fuel use may improve the respiratory health of rural women in Central India. Int J Tuberc Lung Dis.

[24] Sukhsohale ND, Narlawar UW, Phatak MS, Agrawal SB, Ughade SN (2013). Effect of indoor air pollution during cooking on peak expiratory flow rate and its association with exposure index in rural women. Indian J Physiol Pharmacol.

[25] Dutta A, Ray MR (2014). Hypertension and respiratory health in biomass smoke-exposed premenopausal Indian women. Air Qual Atmos Health.

[26] Mukherjee S, Roychoudhury S, Siddique S, Banerjee M, Bhattacharya P, Lahiri T, Ray MR (2014). Respiratory symptoms, lung function decrement and chronic obstructive pulmonary disease in pre-menopausal Indian women exposed to biomass smoke. Inhal Toxicol.

[27] Alim MA, Sarker MA, Selim S, Karim MR, Yoshida Y, Hamajima N (2014). Respiratory involvements among women exposed to the smoke of traditional biomass fuel and gas fuel in a district of Bangladesh. Environ Health Prev Med.

[28] Pial RH, Hashan MR, Ghozy S, Dibas M, El-Qushayri AE, Abdel-Daim MM (2020). Comparative study on respiratory function among rural women using biomass fuel and non-biomass fuel: evidence of a cross-sectional survey in Bangladesh. Environ Sci Pollut Res Int.

[29] Rabha R, Ghosh S, Padhy PK (2019). Effects of biomass burning on pulmonary functions in tribal women in northeastern India. Women Health.

[30] Arora S, Rasania SK, Bachani D, Gandhi A, Chhabra SK (2018). Air pollution and environmental risk factors for altered lung function among adult women of an urban slum area of Delhi: a prevalence study. Lung India.

[31] Mondal NK, Chakraborty D (2015). Vulnerability of rural health exposed by indoor pollution generated from biomass and fossil fuels. Mor J Chem.

[32] Bolla KC, Raghu Y, Jayapalan J, Narasimhan M, Shanmuganathan A, Ganga N (2021). Impact of exposure to biomass fuel on pulmonary function and lung age in rural women. J Evolution Med Dent Sci.

[33] Medgyesi DN, Holmes HA, Angermann JE (2017). Investigation of acute pulmonary deficits associated with biomass fuel cookstove emissions in rural Bangladesh. Int J Environ Res Public Health.

[34] Pratali L, Marinoni A, Cogo A (2019). Indoor air pollution exposure effects on lung and cardiovascular health in the High Himalayas, Nepal: an observational study. Eur J Intern Med.

[35] Adhikari TB, Acharya P, Högman M (2020). Prevalence of chronic obstructive pulmonary disease and its associated factors in Nepal: findings from a community-based household survey. Int J Chron Obstruct Pulmon Dis.

[36] Johnson P, Balakrishnan K, Ramaswamy P (2011). Prevalence of chronic obstructive pulmonary disease in rural women of Tamilnadu: implications for refining disease burden assessments attributable to household biomass combustion. Glob Health Action.

[37] Khalequzzaman M, Kamijima M, Sakai K, Ebara T, Hoque BA, Nakajima T (2011). Indoor air pollution and health of children in biomass fuel-using households of Bangladesh: comparison between urban and rural areas. Environ Health Prev Med.

[38] Nasanen-Gilmore SP, Saha S, Rasul I, Rousham EK (2015). Household environment and behavioral determinants of respiratory tract infection in infants and young children in northern Bangladesh. Am J Hum Biol.

[39] Bates MN, Chandyo RK, Valentiner-Branth P (2013). Acute lower respiratory infection in childhood and household fuel use in Bhaktapur, Nepal. Environ Health Perspect.

[40] Ramesh Bhat Y, Manjunath N, Sanjay D, Dhanya Y (2012). Association of indoor air pollution with acute lower respiratory tract infections in children under 5 years of age. Paediatr Int Child Health.

[41] Khan MS, Lohano HD (2018). Household air pollution from cooking fuel and respiratory health risks for children in Pakistan. Environ Sci Pollut Res Int.

[42] Acharya P, Mishra SR, Berg-Beckhoff G (2015). Solid fuel in kitchen and acute respiratory tract infection among under five children: evidence from Nepal demographic and health survey 2011. J Community Health.

[43] Mandal S, Zaveri A, Mallick R, Chouhan P (2020). Impact of domestic smokes on the prevalence of acute respiratory infection (ARI) among under-five children: evidence from India. Child Youth Serv Rev.

[44] Arlington L, Patel AB, Simmons E, Kurhe K, Prakash A, Rao SR, Hibberd PL (2019). Duration of solid fuel cookstove use is associated with increased risk of acute lower respiratory infection among children under six months in rural central India. PLoS One.

[45] Hasan M, Tasfina S, Haque SM, Saif-Ur-Rahman KM, Khalequzzaman M, Bari W, Islam SS (2019). Association of biomass fuel smoke with respiratory symptoms among children under 5 years of age in urban areas: results from Bangladesh Urban Health Survey, 2013. Environ Health Prev Med.

[46] Karki S, Fitzpatrick AL, Shrestha S (2014). Risk factors for pneumonia in children under 5 years in a teaching hospital in Nepal. Kathmandu Univ Med J (KUMJ).

[47] Mondal D, Paul P (2020). Effects of indoor pollution on acute respiratory infections among under-five children in India: evidence from a nationally representative population-based study. PLoS One.

[48] Nirmolia N, Mahanta TG, Boruah M, Rasaily R, Kotoky RP, Bora R (2018). Prevalence and risk factors of pneumonia in under five children living in slums of Dibrugarh town. Clin Epidemiol Glob Health.

[49] Patel SK, Patel S, Kumar A (2019). Effects of cooking fuel sources on the respiratory health of children: evidence from the Annual Health Survey, Uttar Pradesh, India. Public Health.

[50] Rana J, Uddin J, Peltier R, Oulhote Y (2019). Associations between indoor air pollution and acute respiratory infections among under-five children in Afghanistan: do SES and sex matter?. Int J Environ Res Public Health.

[51] Upadhyay AK, Singh A, Kumar K, Singh A (2015). Impact of indoor air pollution from the use of solid fuels on the incidence of life threatening respiratory illnesses in children in India. BMC Public Health.

[52] Khandker S, Mohiuddin AS, Ahmad SA, McGushin A, Abelsohn A (2023). Air pollution in Bangladesh and its consequences [PREPRINT].

[53] Naz S, Page A, Agho KE (2018). Potential impacts of modifiable behavioral and environmental exposures on reducing burden of under-five mortality associated with household air pollution in Nepal. Matern Child Health J.

[54] Muhumuza R, Zacharopoulos A, Mondol JD, Smyth M, Pugsley A (2018). Energy consumption levels and technical approaches for supporting development of alternative energy technologies for rural sectors of developing countries. Renew Sustain Energy Rev.

[55] Behera B, Mallick B (2023). Constraints perceived by dealers and households for execution and adoption of Pradhan Mantri Ujjwala Yojana. Indian J Extension Educ.

